# Establishment of a condition-specific quality-of-life questionnaire for children born with esophageal atresia aged 2–7 across 14 countries

**DOI:** 10.3389/fped.2023.1253892

**Published:** 2023-10-23

**Authors:** The International EA-QOL Group, Michaela Dellenmark Blom

**Keywords:** esophageal atresia, quality of life, translation, validity, cognitive debriefing, rare disease, children

## Abstract

**Background:**

Esophageal atresia (EA) is a rare congenital anomaly characterized by a discontinuity of the esophagus. Following surgical repair, survival rates have improved dramatically the past decenniums and today exceed 90%, but the children commonly present with esophageal and respiratory morbidity. In 2018, a condition-specific quality-of-life questionnaire for children with esophageal atresia (EA) aged 2–7 in Sweden-Germany was finalized (The EA-QOL questionnaire). The study aim was to describe the evaluation of the new translations across 12 new countries in Europe, Asia, Africa, Central-and North America.

**Methods:**

Following forward-backward translation into the new languages, the 17-item EA-QOL questionnaire was tested in cognitive debriefing interviews with parents of children with EA aged 2–7. Parents rated if each item was easy to understand (clarity) and sensitive to answer (interference with personal integrity). They could skip responding to a non-applicable/problematic item and give open comments. Predefined psychometric criteria were used; item clarity ≥80%/item sensitive to answer ≤20%/item feasibility ≤5% missing item responses. The decision to modify the translation was based on native expert, patient stakeholder, and instrument developer review, and the need for harmonization between translations.

**Results:**

Similar to findings in the Swedish-German cognitive debriefing, the cross-cultural analysis of input from 116 parents from 12 new countries (4–14 parents, median 9 parents/country) showed that all items in the EA-QOL questionnaire fulfilled the criteria for item clarity ≥80% and sensitive to answer (ranging from 1%-4.5%), although results varied between countries. Four items had missing responses between 5.2% and 13.4%, three within the same domain and were in line with parents’ explanations. Poor translations and feasibility were improved.

**Conclusions:**

Based on parent input, the collaboration between native experts, patient stakeholders, and instrument developers, a linguistic version of the EA-QOL questionnaire for children aged 2–7 for use in and across 14 countries has been established. These efforts have set the conditions for a cross-cultural field test of the EA-QOL questionnaire and will open the doors for a new chapter in outcome research, registries, and clinical practice concerning children with EA. In the long-term, this will help increase knowledge of the disease's burden, promote patient-centeredness, exchange of information between nations, and strengthen evidence-based treatments for children born with EA.

## 1. Introduction

**Figure 1:**
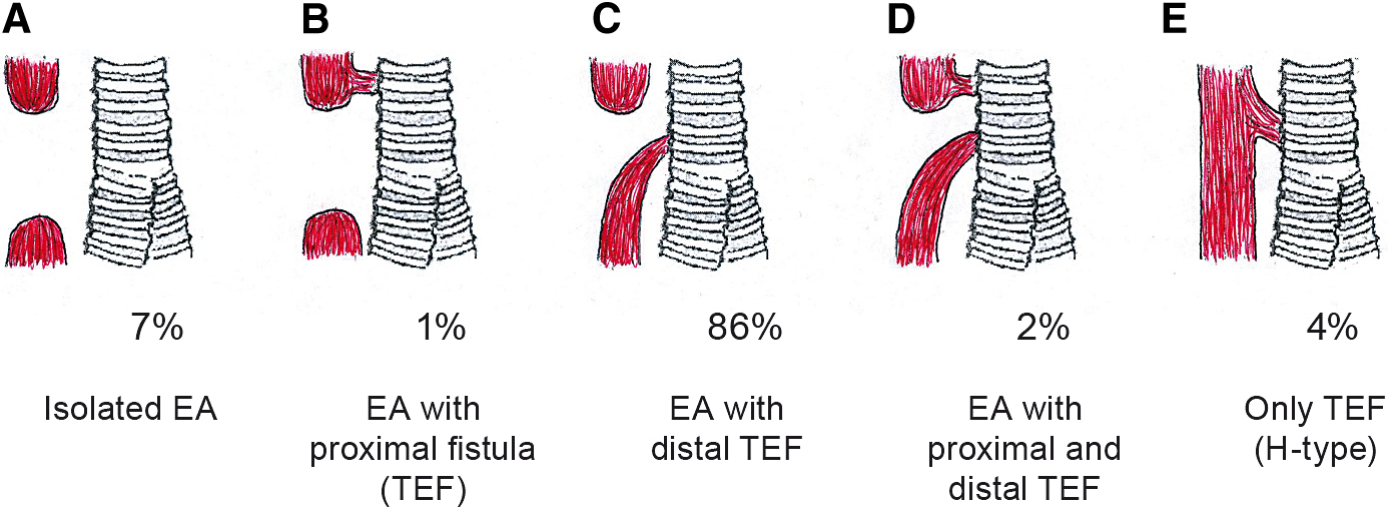
Presentation of subtypes of esophageal atresia according to the Gross classification system, and their prevalence. The red color illustrates the esophagus, and the gray color represents the windpipe. Gross A; interrupted esophagus without any connection to the windpipe. Gross B; interrupted esophagus with a connection to the windpipe from the upper (proximal) esophageal segment. Gross C; interrupted esophagus with a connection to the windpipe from the lower (distal) esophageal segment. Gross D; interrupted esophagus with a connection to the windpipe from both the proximal and the distal esophageal segments, and Gross E/H-type refers to a connection to the windpipe without any interruption of the esophagus. The illustration is reprinted with permission from Vladimir Gatzinsky.

Esophageal atresia (EA) is a rare congenital anomaly characterized by a discontinuity of the esophagus. It presents in different anatomical subtypes in relation to the presence and/or location of a tracheoesophageal fistula (Figure 1). EA co-occurs with other anomalies, most frequently of the cardio-vascular, uro-genital, and digestive system (1, 2). In most children, a primary esophageal repair can be accomplished within the first days of life (3). Today more than 90% of the children survive (4), but they commonly present with dysphagia (43%–71%) (5), anastomotic strictures with a need for dilatation (58%), gastroesophageal reflux disease (44%–65%) (5–7) and feeding difficulties (63%) (8). Respiratory symptoms are also frequent (52%–69%), including chronic and/or barking cough, wheezing, recurrent respiratory infections, and dyspnea (9, 10). Some children may also suffer from poor somatic growth retardation (11). This morbidity may be more pronounced during the first years of life (11–13). The children's access to multidisciplinary follow-up care varies between and within countries (14–16).

In recent years, recommendations for different care and treatment of patients with EA have been published by several expert stakeholders; the European and North-American Society for Paediatric Gastroenterology Hepatology and Nutrition (17), the International Network for EA (INoEA) (9) and the European Reference Network for rare Inherited and Congenital Anomalies (ERNICA) (18), which proclaim the need for health care providers to focus on the patients’ health-related quality of life (HRQOL).

HRQOL refers to the individual's perception of the impact of disease and treatment on physical, social, and psychological functioning and well-being and can be measured using generic or condition-specific questionnaires (19). Research of HRQOL in children with EA has grown in the last past years (20, 21), inconsistently showing that their generic HRQOL is comparable (22–24), better (25, 26) and worse (27–29) compared to general populations. The purpose of a condition-specific HRQOL questionnaire is to measure aspects of relevance to the specific population and clinical context. In 2018, a set of age-specific condition-specific HRQOL questionnaires for children with EA in Sweden and Germany was developed (The EA-QOL questionnaires) according to international recommendations for patient-reported outcome measurements (PROMs) (30–33). Items were generated based on focus groups with children and their parents, allowing adjustment of item content and wording for child age (31). The subsequent versions for children aged 2–7 (parent- report) and children aged 8–18 (self-and parent-report) were psychometrically evaluated for families of children with EA (34–36).

A person's perception of a HRQOL questionnaire may be influenced by norms, values, and standards embedded within their country and language (31, 37, 38). Recommendations for translation and cultural adaptation of a HRQOL questionnaire are available to aid a conceptually and semantically equivalent version of an HRQOL questionnaire that is understood by its target population across different countries (39, 40). To date, the EA-QOL questionnaire for children with EA aged 2–7 has been field tested in Sweden and Germany (36), Turkey (41), Poland (42), and the Netherlands (43). However, in the past five years, the number of countries involved in the translation and psychometric evaluation has increased significantly. This development prompted an investigation of the cross-cultural applicability of the EA-QOL questionnaires, as it carries the potential for children with a rare disease like EA to have standardized outcome assessments for use in research and clinical practice (44). The study aimed to describe the establishment of the EA-QOL questionnaire for children born with EA aged 2–7 for use in and across 14 countries, following evaluation of its linguistic and content validity as well as feasibility, prior to commencing a cross-cultural field test.

## 2. Material and methods

### 2.1. Translation and evaluation of the EA-QOL questionnaire in additional languages/countries

**Figure 2:**
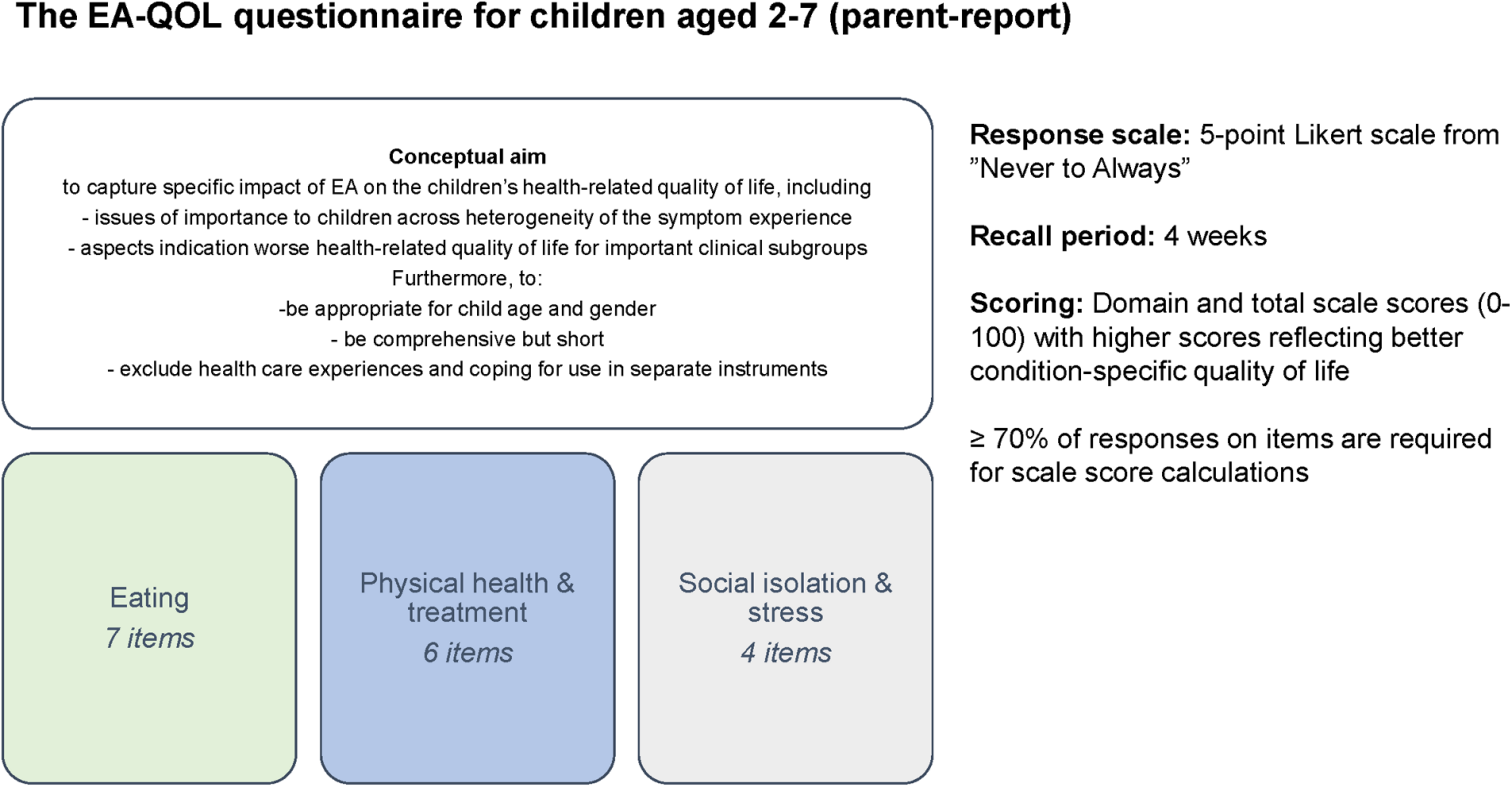
The conceptual aim and structure of the EA-QOL questionnaire for children aged 2−7.

Figure 2 presents the conceptual aim and structure of the EA-QOL questionnaire for children aged 2–7. After this questionnaire was finalized for use in Sweden and Germany in 2018 (36), it was licensed by 14 researchers interested in translating and evaluating its psychometric properties in their country, together with a study protocol describing this procedure. The study protocol aimed to support semantical/conceptual equivalence of the translations of the EA-QOL questionnaire and standardize the psychometric evaluation across countries, and was guided by recommendations for PROMs from the International Society for Pharmacoeconomics and Outcomes Research (ISPOR) and US Food and Drug Administration (30, 32, 33, 39, 45). Between June and October 2021, the researcher responsible for the study in their country was invited to the first joint international EA-QOL initiative by the study coordinator in Sweden (MDB), thirteen of whom accepted participation. The researchers functioned in Africa (South Africa), Asia (China), Europe (Croatia, France, Germany, Hungary, Norway, Poland, Spain, Turkey, United Kingdom-UK), North America (USA), and Central America (Mexico). In each country, the research team involved native specialists in the field of EA. Additionally, three patient stakeholders from EAT (GS, AWG, VW), global support group associated with EA, were invited to include patient perspectives in this study.

### 2.2. Definitions and framework for evaluation standards

The definitions of linguistic validity, content validity, and item feasibility outlined in Table 1 were used in the evaluation of the EA-QOL questionnaire (32, 33, 39, 45–47). Furthermore, the Swedish-German evaluation of the EA-QOL questionnaire (35, 36) was employed as a framework for the additional language versions, as it gave rise for the primary item evaluation.

**Table 1:** The definitions of linguistic validity, content validity and item feasibility used in the evaluation of the EA-QOL questionnaire for children aged 2−7.

Linguistic validity	A multi-stage process needed to ensure that the instrument had been correctly translated into the target language considering clarity, appropriateness, and cultural relevance, and to ensure that the translation stated in the target language what the original in the source language intended
Content validity	Evidence that the instrument measured the concept of interest, including evidence that the items and domains of the instrument were appropriate, understandable and comprehensive relative to its intended measurement concept, population and use
Item feasibility	The respondents can reply to an item and provide complete data in future applications of the EA-QOL questionnaire

### 2.3. Forward-backward translation

**Figure 3:**
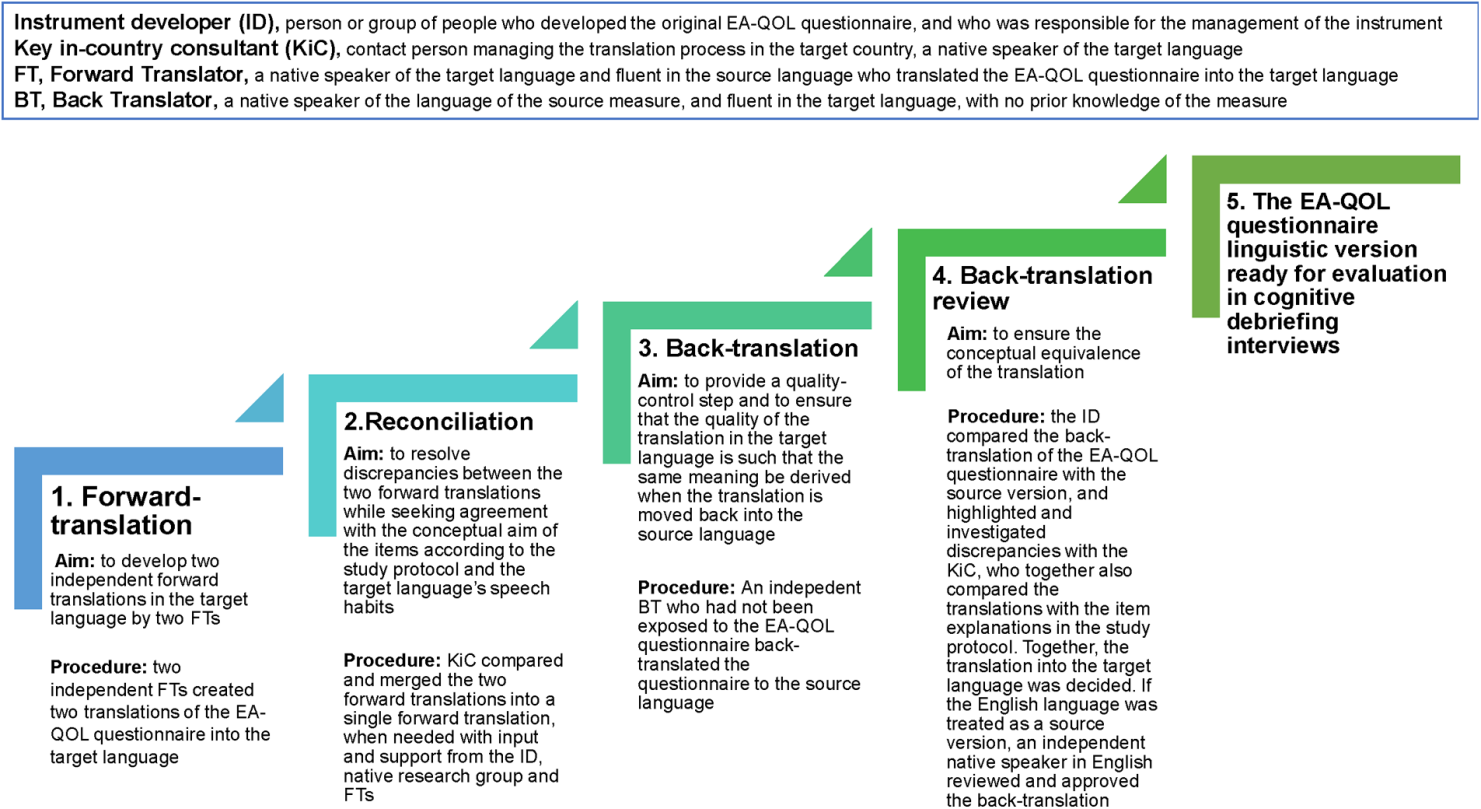
The translational procedure of the EA-QOL questionnaire.

The translational procedure of the EA-QOL questionnaire is presented in Figure 3. Following the Swedish-German finalization in 2018 (35, 36), the EA-QOL questionnaire was translated into 11 further languages for testing in 12 countries. Supplementary Material 1 gives an overview of the languages/countries, year of translation, professionals involved in the forward-backward translation, results of the back-translation review, and describes the procedure in more detail.

## 2.4. Cognitive debriefing

### 2.4.1. Study participants

Parents of children aged between 2 and 7 who were born with EA Gross type A-E and were residents in the target country were invited to participate in a cognitive debriefing interview of the translated EA-QOL questionnaire by a local researcher. In Sweden and Germany, cognitive debriefing interviews were conducted with 16 parents of children with EA (child median age 4 years, 12 boys, 12 Gross type C, 10 children resident in Germany) (35). Similarly, the study protocol for testing the EA-QOL questionnaire in new countries proposed a small number of parents of children to be recruited, with different severity of disease, as described earlier (42, 43, 48). In each of the 12 additional countries, 4–14 parents (median 9) participated in the cognitive debriefing of the EA-QOL questionnaire; in total, 116 parents of 113 children with EA (Table 2). The parents were mostly mothers (86.2%), whose children commonly had EA Gross type C (84.1%), and slightly more than half of the children (56.6%) were male.

**Table 2:** Characteristics of the study sample of children born with esophageal atresia and their parent-proxies in 12 new countries where the translated EA-QOL questionnaire was tested in cognitive debriefing interviews.

	Cross-cultural characteristics *n* (%)
Child information (*n* = 113)	
Child male sex	64 (56.6)
Gross type ^a^	
A	14 (12.4)
B	2 (1.8)
C	95 (84.1)
Child age (median/min-max)	4 (2–7)
Country of residence	
Croatia	14 (12.4)
USA	13 (11.5)
Mexico	13 (11.5)
United Kingdom	11 (9.7)
Norway	10 (8.8)
Hungary	9 (8.0)
Poland	9 (8.0)
Spain	8 (7.1)
Turkey	8 (7.1)
China	8 (7.1)
France	6 (5.3)
South Africa	4 (3.5)
Parent information (*n* = 116)	
Mother	100 (86.2)
Parent age (years)	35 (20–48)^b^

^a^ 2 missing values.

^b^ 4 missing values.

### 2.4.2. Data collection

**Figure 4:**
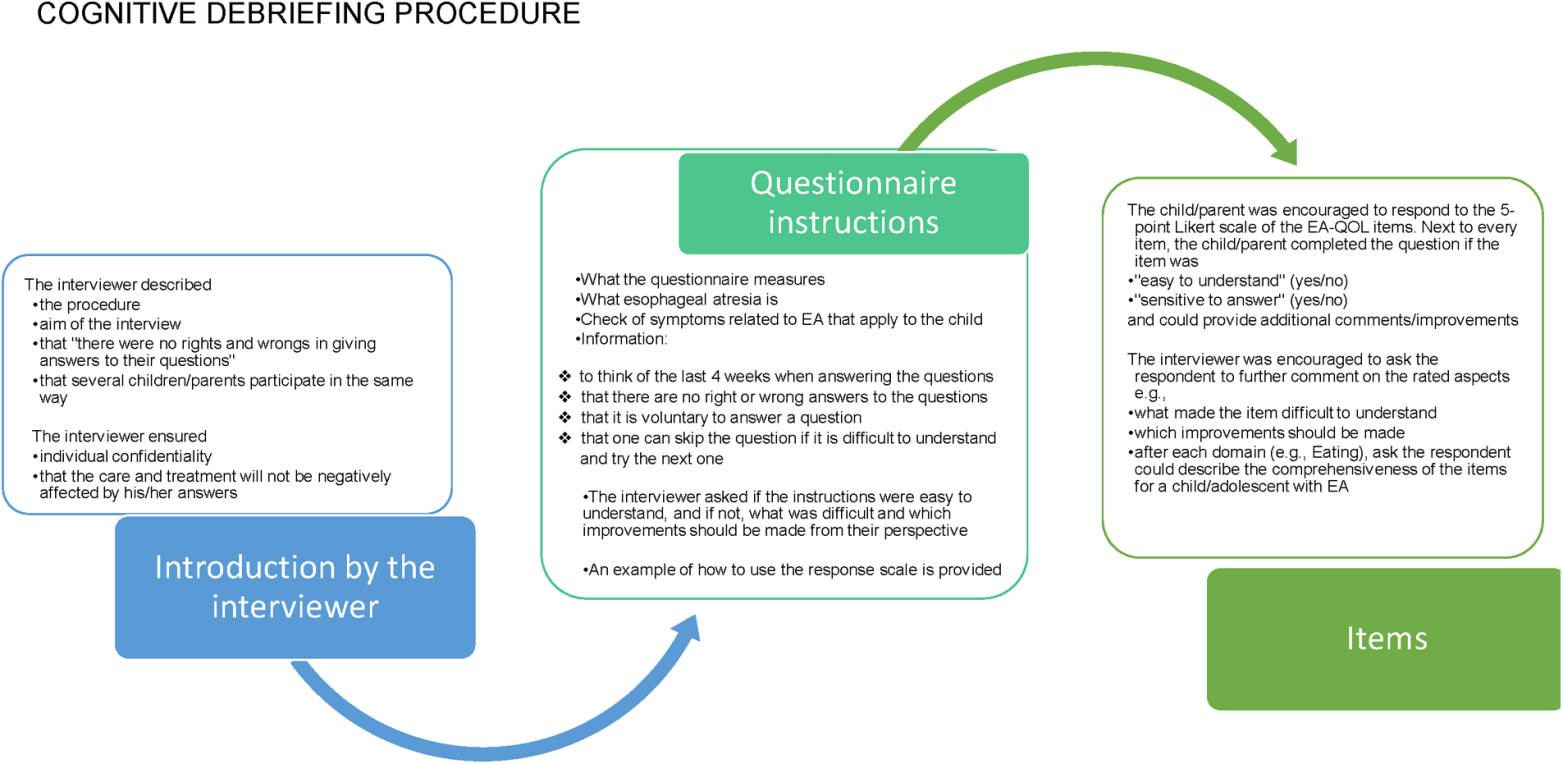
The cognitive procedure used in the evaluation of the EA-QOL questionnaire for children aged 2−7.

Supplementary Material 2 presents the data collection method used in the cognitive debriefing of the EA-QOL questionnaire. In nine countries, the study participants were recruited by convenient and purposive sampling from clinical centers’ follow-up programs only. In the UK, study participants were recruited from a patient support group (TOFS). In Norway and Hungary, both recruitment sources were used. In 11 countries, the parents were interviewed by a researcher with a healthcare professional background, and in nine countries, interviews were held at the clinical center/hospital. The procedure for the cognitive debriefing is outlined in Figure 4. During the interview, the researcher made field notes of the respondent's comments on the EA-QOL questionnaire, which were translated into English by a local researcher. All cognitive debriefing responses were registered in an excel-file with basic characteristics of the respondent (child sex, child age, Gross type EA, parent gender, and age).

### 2.4.3. Data analysis

Statistical data were analyzed using IBM SPSS Statistics for Windows (Version 25.0, Armonk, NY, USA: IBM Corp). The study population was analyzed using descriptive statistics. All items in the EA-QOL questionnaire were analyzed regarding item clarity (yes/no), sensitivity to answer (yes/no), and item missing responses (*n*, %) on a country-specific and accumulated cross-cultural level. As the Swedish-German data (35) was regarded as a framework, it was excluded from the cross-cultural evaluation of the newly translated items.

A country-specific researcher first sorted the comments made by parents from into positive/confirmative comments and negative/difficult comments for each item. Then, the instrument developer (MDB) used manifest content analysis (49), including an inductive bottom-up categorization process, across all countries to define the types of item difficulties reported by parents. One statement/comment could only be sorted into one category, but one parent could give statements that matched different categories. Each country-specific research team then reviewed the suggested categories generated from their study material. Based on this feedback, the instrument developer (MDB) decided on the cross-cultural categorizations to be reviewed, discussed, and finalized with two methodologists (JHQ, SW). For each category, the number and percentage of respondents were estimated. Furthermore, the respondents’ comments on the questionnaire instructions and response scale were listed.

Table 3 presents the psychometric criteria serving as indication for the need of rewording and/or adjusting the translated item in the EA-QOL questionnaire for children aged 2–7 for conceptual/linguistic/cultural appropriateness in the specific country. These criteria were also used in the initial Swedish-German evaluation of the EA-QOL questionnaires (35, 36) and included item clarity ≥80%, item sensitive to answer ≤20%, item feasibility ≤5% and strength and difficulties reported by children and/or their parents.

**Table 3:** Psychometric criteria serving as indication for the need of rewording and/or adjusting the item in the EA-QOL questionnaire for children aged 2–7 for conceptual/linguistic/cultural appropriateness in the specific country.

Criteria for item performance	Item clarity ≥80%	Item sensitive to answer ≤20%	Item feasibility ≤5%	Strength and difficulties reported by children and/or their parents
Interpretation	If an item was easy to understand, this indicated that a translation in the new country was satisfactory, the respondent recognized/understood the item content in context of EA (ie familiar with the possible problem) and could provide good data in future applications of the questionnaire	If an item was sensitive to answer, this indicated that a respondent may find it difficult to provide an open reliable answer when it interferes with personal integrity	If there was a high proportion of missing item responses, this indicated that an item may be problematic which should call for attention and possible revision	Combined statistical and qualitative findings in small samples were required when interpreting the item performance. If similar difficulties or suggestions for improvement of the item were raised by study participants, careful note should be taken by the research team.

NA, not applicable.

#### 2.5. Harmonization between different language versions of the EA-QOL questionnaire

The harmonization process was conducted by the country-specific study coordinator(s) and instrument developer (MDB) to detect and deal with translation discrepancies between different language versions of the EA-QOL questionnaire, thus ensuring conceptual equivalence between the source and target language versions and between all translations, as well as justification of cultural adaptations (39, 45). The primary instrument developer (MDB) had been a part of establishing each translation, reviewed all back-translations and brought these experiences into the harmonization process. The solutions for harmonization were shared at any point during the study process, however, for the same language employed in several countries (USA- UK- South Africa and Spain-Mexico respectively), the translations were compared and harmonized *after* their respective cognitive debriefing interview study, with the help of native experts (BZ, ND, SE, CdV, JDHP, ASG), the instrument developer (MDB) and patient representative (GS). Similarly, after the cognitive debriefing studies, the Chinese Mandarin and Hungarian versions of the EA-QOL questionnaire were again reviewed against the US-UK English version of the EA-QOL questionnaire by the native experts (China SL, Hungary KM) and the instrument developer (MDB).

#### 2.6. Modifications/changes of the translations and/or the EA-QOL questionnaire

Decision on the need to modify/improve item wording of a translation was made based on cognitive debriefing results, review by native experts and instrument developer, and the need for harmonization between languages. The decision on the need to modify the EA-QOL questionnaire cross-culturally was based on international item performance (32, 33, 39, 50), was discussed with all country-specific research teams and EAT representatives, and ultimately decided by the instrument developers (MDB, JD, JQ, SW).

## 3. Results

Supplementary Material 3 details information of item performance from cognitive debriefing of the EA-QOL questionnaire conducted with parents from Europe (Sweden-Germany, Croatia, France, Hungary, Norway, Poland, Spain, Turkey, UK), Africa (South Africa), Asia (China), Central America (Mexico) and North America (USA). Table 4 presents the cross-cultural results of the cognitive debriefing of the 17 translated items of the EA-QOL questionnaire conducted with parents of 113 children with EA from 12 countries.

**Table 4:** The cross-cultural results of the cognitive debriefing with parents of 113 children with born with esophageal atresia in 12 countries.

Domains		Items	CROSS-CULTURAL		
			Easy to understand^a^	Sensitive to answer^a^	Missing item responses^b^
Eating	1.	It is difficult for my child to eat age-appropriate food because food sticks in their throat	110 (97.3)	1 (0.9)	0
	2.	It is difficult for my child to eat a full meal	105 (92.9)	2 (1.8)	1 (1.0)
	3.	Eating stresses my child	109 (97.3)^c^	3 (2.7)	0
	4.	My child can eat at the pace they want	106 (93.8)	1 (0.9)	0
	5.	My child is worried when they choke on food	104 (92.0)	3 (2.7)	0
	6.	It bothers my child when they vomit	104 (92.0)	3 (2.7)	0
	7.	My child requires certain adaptations so they can eat food at a party or when out with friends	108 (95.6)	5 (4.5)^a^	1 (1.0)
Physical health & treatment	8.	My child gets tired easily when they play games or sports	113 (100)	3 (2.7)	0
	9.	My child has less strength than other children during physically demanding activities	109 (96.5)	3 (2.7)	0
	10.	My child is bothered by respiratory problems (e.g. coughing, phlegm, or difficulty breathing)	104 (92.0)	3 (2.7)	1 (1.0)
	11.	It is a problem for my child that my child gets respiratory infections easily	93 (83.0)	1 (0.9)^c^	5 (5.2)
	12.	My child hates taking medicine	104 (92.0)	2 (1.8)^d^	4 (4.1)
	13.	My child's health condition makes it difficult for them to fall asleep or stay asleep at night (e.g. reflux, coughing, anxiety)	110 (97.3)	1 (0.9)	0
Social isolation & stress	14.	Preschool/school absence due to my child's health condition impacts my child's life negatively	94 (87.9)^e^	3 (2.9)^h^	13 (13.4)
	15.	It is hard for my child to explain to others what they can and cannot do	101 (92.7)^f^	3 (2.8)^i^	12 (12.4)
	16.	It bothers my child that people make comments about them (e.g. coughing, scars, choking)	103 (93.6)^g^	4 (3.7)^f^	8 (8.2)
	17.	It bothers my child that people react negatively when they make a noise (e.g. breathing, clearing his/her throat, coughing,, wheezing)	105 (94.6)^d^	4 (3.6)^d^	4 (4.1)

^a^ Parents of 113 children from Croatia, USA, Mexico, United Kingdom, Norway, Hungary, Poland, Spain, Turkey, China, France, South Africa.

^b^ Parents of 97 children from Croatia, USA, Mexico, United Kingdom, Hungary, Poland, Spain, Turkey, France, South Africa.

^c^ 1 missing value.

^d^ 2 missing value.

^e^ 6 missing value.

^f^ 4 missing value.

^g^ 3 missing value.

^h^ 8 missing value.

^i^ 5 missing value.

### 3.1. Item clarity

As in the Swedish-German cognitive debriefing (35), the cross-cultural analysis from 12 countries showed that all items in the EA-QOL questionnaire for children aged 2–7 fulfilled the criteria for item clarity. In fact, 15/17 translated items were rated as easy to understand by >90% of the parents. In seven countries (China, Croatia, Hungary, Mexico, Poland, South Africa, and Turkey), all 17 items fulfilled the criteria for item clarity, as 92.3%–100% of the parents rated the translated items as easy to understand. In three other countries (France, Spain, USA), two items each did not achieve the desired level of clarity (France: items 11 and 12; Spain: items 6 and 10; USA: items 5 and 11). In two other countries, six items (UK: items 1, 6, 10, 11, 13, and 14) and seven items (Norway: items 2, 5, 12, and 14–17) did not fulfill the desired criteria (Supplementary Material 3).

### 3.2. Item sensitive to answer

Similar to the Swedish-German cognitive debriefing (35), the cross-cultural analysis from 12 countries revealed that all items in the EA-QOL questionnaire fulfilled the criteria as only a few parents (ranging from 1%–4.5%) rated the items as sensitive to answer. In 11 countries, all items achieved the predefined criteria, while in one country (South Africa), one out of totally four parents rated four items as sensitive to answer (items 3, 6, 7 and 17).

### 3.3. Item feasibility

In contrast to the Swedish-German cognitive debriefing, cross-culturally, 13/17 items achieved the desired level of feasibility. The remaining four items (items 11, 14, 15, and 16) had missing responses varying between 5.2% and 13.4%, three of which were found within the Social isolation & Stress domain (items 14, 15, and 16). A detailed analysis revealed that missing item responses for item 14 were, at some level, found across the six countries Croatia, France, Hungary, South Africa, the UK, and the USA. Out of 13 missing item responses, 11 came from parents of children aged <4 years. Similar patterns of missing items responses were found across the four countries, France, Hungary, South Africa, and the UK, regarding item 15 and item 16; all came from parents of children aged <4 years.

### 3.4. Comments from parents

Supplementary Material 4 shows categories of parents’ understanding and perceived difficulties of items in the EA-QOL questionnaire for children with EA aged 2–7 identified through parents’ comments. The number of parents who described their conceptual understanding of an item or their perceived type of item difficulty is presented. Out of 116 parents, the items received comments by a subsample; recognition/understanding of an item included at the most comments from 12 parents, and an item difficulty category included at the most comments from 16 parents. The definition of each category is presented in Table 5.

**Table 5:** Category, explanation, and example of comments to the items in the EA-QOL questionnaire, made by parents of children with esophageal atresia.

Category	Explanation	Example of comment
Recognition and conceptual understanding of the item	• Conceptual understanding of the item content in the context of EA • Confirmation in open replies that the item was easy to understand	If she eats too fast, food gets stuck, (item 4, parent from South Africa)
Unclear/ambiguous wording	• Unclear translation • Ambiguous term/phrasing • Did not recognize the item issue in context of EA • Similarity between items	Clarify that question ask if vomiting cause the child distress/bothers them unsure of problem physically, ie it occurs (item 6, parent from United Kingdom)
Difficult to answer the item if no current experience of the symptoms/the situation	• No experience of the symptom or situation within the recall period	My child did not experience choking (item 5, parent from Hungary)
Difficult to answer due young child age <4 years	• Not applicable due to young child age	The child is too young to be bothered by reactions of other people (parent from Croatia)
Parents/the family were bothered by the problem rather than or additional to the child	• Parents described that they/the family were bothered instead of or additionally to their child	It's a problem for me but not for my child even though he is often absent from school (parent from France)
Emotive question/strong expression	• Item wording is too emotive and/or can be upsetting	Word frightened—strong and emotive (parent from UK).

#### 3.4.1. Recognition and conceptual understanding of the item

Across various countries, all 17 items received feedback from parents regarding their conceptual understanding of the item in the context of EA or their confirmation that the item was easy to answer. Most frequently (>10 parents), this was described in relation to four items within the eating domain (items 1, 4, 6, and 7) and three items within the Physical health & Treatment domain (items 10, 12, and 13).

#### 3.4.2. Perceived item difficulties

Parents’ comments on item difficulties mostly considered either unclear/ambiguous wording or difficulties to answer an item if the child had no current experience of symptoms/the situation.

Cross-culturally, at least one parent in several countries mentioned why the translation of four items within the eating domain was unclear/ambiguous (item 1; Croatia, UK/item 4; UK, Spain, Norway, Mexico/item 5; China, UK, Norway/item 6; Croatia, UK, Hungary, Norway, Mexico) or difficult to answer item 6 as the child had no current vomiting problems and/or had undergone antireflux surgery (Croatia, Hungary, UK, Turkey, Spain). Eight parents described the translations of item 10 (UK, Spain, Croatia), and nine parents the translations of item 12 (China, France, Norway) within the Physical health & Treatment domain as unclear/ambiguous. Furthermore, in agreement with patterns of missing item responses, between 5 and 13 parents of children aged <4 years from six countries (Croatia, Hungary, Norway, UK, South Africa, and Spain) commented on difficulties to answer items within the Social isolation & Stress domain, (items 14–17) because the child was too young. Sixteen parents also described that their child had not yet started school which interfered with the feasibility of responding to item 14.

Considering specific languages and corresponding to ratings of item clarity, parents from:

• Norway gave input as to why the translation of items 2, 5, and 12 was unclear and between 2 and 4 parents explained that it was difficult to answer items 14–17 due to young child age (2–3 year olds)
• The UK commented that the primary English translation of items 6, 7, 10, 11, and 14 was unclear as to whether “the symptom occurred/happened or bothered the child”
• France gave input as to why the wording of item 11 was inappropriate and suggested improvements
• China provided their view of the term “choke” (item 5), which had two meanings in Chinese Mandarin language, and said they preferred the translation referring to “cough caused by inhalation of food into the trachea while eating” (48).

#### 3.4.3. Item comprehensiveness

The following aspects were mentioned to enhance item comprehensiveness; family impact (a parent each from Hungary and France), gastrostomy (a parent from France), oral sensory issues such as the impact on brushing teeth (a parent from France), and esophageal dilatation (a parent from Hungary). Furthermore, it was described that for children with EA and concomitant anomalies, a condition-specific questionnaire for EA might not capture the holistic situation as other anomalies may also impact their HRQOL (parents in Norway).

### 3.5. Questionnaire instructions

The questionnaire instructions had complaints by parents from one country (Norway) because it contained too much and too difficult text.

### 3.6. Response scale

The option of adding “non-applicable” to the 5-point Likert response scale was suggested by study participants or experts in five countries to improve the applicability of the response options (the UK, Norway, Croatia, Hungary, and the US).

### 3.7. Modifications/changes of the translations and/or the EA-QOL questionnaire

Table 6 presents an overview of the translated items of the EA-QOL questionnaire which did not achieve the desired psychometric criteria and those which were improved in wording. A description of the process is detailed in Supplementary Material 5. It reveals that the harmonization process led to a linguistically equivalent UK- US English version and European-Mexican Spanish version of the EA-QOL questionnaire. Furthermore, as items 14–17 of the Social isolation & Stress domain did not fulfill the predefined criteria for feasibility for children aged <4 years in 4–6 countries with a similar tendency observed in the Swedish-German evaluation (36, 51), the instrument developers decided to increase the child age to 4 years for this domain and thereby improve the cross-cultural applicability (see Table 6 and Supplementary Material 5).

**Table 6:** Presentation of changes/modifications made in item wording based on the cognitive debriefing, review by experts, instrument developer and patient stakeholders and need for harmonization of the translated EA-QOL questionnaire for children aged 2–7.

Country	Items which did not achieve the desired criteria				No change of the translated items	Changes in item wording per scale/domain			Conceptual cross-cultural change
	Item clarity^a^	Item sensitive to answer^b^	Item feasibility^c^			Eating (item 1–7)	Physical health & treatment (item 8–13)	Social isolation & stress (items 14–17)	Social isolation & stress
Turkish				Decision	X				Items 14–17 will be answered only by parents of children born with esophageal atresia aged 4–7
Polish					X				
Hungarian			14,15,16,17		X				
Croatian			14		X				
French	11,12		14,15,16				11, 12		
Norwegian	2,5,12,14,15,16,17					2, 5, 6			
China						1, 5, 7			
US English	5,11		14			1, 6, 7	13	14, 16, 17	
UK English	1,6,10,11,13,14		14,15,16,17			1, 6, 7	13	14, 16, 17	
South African English		3,6,7,17	14,15,16			1, 2, 3, 4, 6	9, 10, 11, 13	14, 16, 17	
European Spanish	6,10					1,3,4,5,6	9,11	15,16	
Mexican Spanish						1,2,5	8,10,11,12,13	15,16	

^a^ Item clarity ≥80%.

^b^ Item sensitive to answer ≤20%.

^c^ Item feasibility ≤5%, included data from Croatia, France, Hungary, Poland, Spain, Turkey, United Kingdom, South Africa, Mexico and USA, ie data from Norway and China were excluded in this study.

## 4. Discussion

This study presents the establishment of a linguistic version of the EA-QOL questionnaire for children aged 2–7 for use in and across 14 countries from Africa, Asia, Europe, Central America, and North America, following translation, cognitive debriefing, expert, patient stakeholder and instrument developer review and harmonization between languages.

### 4.1. Translation

Our study reflects the work of 22 forward translators, 11 back-translators, 12 native specialist teams, and six authors who originally developed the EA-QOL questionnaires. Translating an instrument into another language is a formidable and resource-demanding task (52), yet, translation remains the most crucial step in adopting an instrument for use in another country. Careful work is necessary to unify the conceptualization of the studied phenomenon across different languages and enable successful future aggregation of international data sets, which is critical to achieving the benefits associated with an increased sample size (39, 52). This is especially important in a rare disease like EA. In response, international and collaborative studies are increasing in this field, including by stakeholders like ERNICA (18), INoEA (17), as well as the patient federation EAT (53). It took five years after the EA-QOL questionnaires were finalized in Sweden and Germany (36) to establish appropriate linguistic versions in 12 new countries, indicating an emerging chapter of multinational outcome research in children with EA.

The primary item generation of the EA-QOL questionnaire was developed in Swedish (34), a North Germanic language spoken predominantly in Sweden (10 million people) (55). Despite being a low-population country, a systematic review from 2022 showed that Sweden was the second most common country to conduct PROM studies in pediatric surgery (44). Still, in comparison, most translation experience relies on therein that HRQOL instruments have generally been developed in USA or UK in English (56, 57). The translation of the EA-QOL questionnaire was guided by steps outlined by ISPOR (39) with key elements such as the key-in-country person, involvement of the instrument developer, and a study protocol with a list of explanations of the items available during forward-backward translation (39). Nevertheless, there was variation between countries in the translation procedure concerning the source of recruitment for translators, language, and time point of the translation, which reflects resources and bilingual translators available in each setting. Full compliance of the ISPOR guidelines may be difficult for condition-specific questionnaires which are being internationally adapted (58), especially rare diseases face particular challenges (59). For example, our study lacked professional translators which may influence the findings.

The new translations of the EA-QOL questionnaire aimed to maximize the attainment of semantic and conceptual equivalence with the original source version (37, 39) rather than being a literal translation which is recommended for more subjective constructs like HRQOL (39). If this is not achieved, the instrument will be less likely to maintain the psychometric performance that the source measure demonstrated (37, 39). In our case, between 1 and 8 items were resolved for semantical equivalence after the back-translation review (Supplementary Material 1), with close attention paid to an agreement with the original version. Modification of items after back-translation review is found in other instruments, and the degree of inconsistencies may vary by country/language and measurement areas (58, 60, 61).

### 4.2. Cognitive debriefing

As recommended, the new translations of the EA-QOL questionnaire were evaluated in cognitive interviews regarding all its components (30, 33, 39). This is critical to the instrument's content validity since it offers adjustment in the measure before it is administered for testing in a larger psychometric evaluation study (39), such as a cross-cultural field test. This study employed cognitive debriefing interviews to identify and resolve unclarity or inadequacy in wording or cultural appropriateness of a translated questionnaire (32, 33, 50). Culture is a complex term involving political, geographical, anthropological, sociological, and psychological aspects connected to beliefs and values that give life meaning and purpose (37). Although cognitive debriefing interviews are limited to capturing the whole complexity of culture, such aspects are said to be reflected in the notion of language, which is why a translation should aim to respect the normal speech patterns and colloquialisms of the target country-culture (39, 45). Hence, this procedure also evaluated translation alternatives that the translators might not have resolved by consulting parents, like for the term “choke” in Chinese Mandarin (48).

We found that most parents rated the items as easy to understand, which agrees with other cognitive debriefing results of PROMs completed in multiple countries (58, 62). However, on country-level our study revealed that the English and Norwegian languages required most improvements of the translations. Compared to previous literature, it is debated whether an HRQOL questionnaire is conceptually and linguistically transferable between East and West (56), but this was not challenging for the EA-QOL questionnaire (48). Regarding the English language, a reason may be that the initial translation was literal rather than semantical, despite carrying out careful translation procedures. Therefore, there was a need for adaptation of the items to the speech habits of the English-speaking target population. In Norwegian, items within the Social isolation & Stress domain were challenging to answer for parents of the youngest children. In this view, these items also had poor feasibility across several countries for children aged <4 years. Considering the content, the translation of item 14 (the child's preschool/school absence) most consistently lacked clarity. In response to this, the conceptual structure of the EA-QOL questionnaire for children aged 2–3 years was adjusted in agreement with the ISPOR recommendations (31), stating that child age and differences in the educational system and social activities in children between countries need consideration in PROM development. This decision was made to increase the future completeness of data when using the EA-QOL questionnaire and generalizability of study findings. The EA-QOL questionnaire was initially developed cross-culturally in two North European countries (35, 36). Should the initial item evaluation have included a larger cross-cultural sample, this may have become apparent earlier in the study process, which indicates the benefits of a simultaneous cross-cultural approach.

Our cognitive debriefing also included analyzing whether an item was sensitive to answer, to understand its perceived interference with personal integrity (50). Not only can sensitive items interact with openness of parents’ replies (50), but a questionnaire should be well received among the target population (50, 63). Interestingly, cross-culturally, parents in this study did generally not rate the items of the EA-QOL questionnaires as sensitive to answer. A possible reason may be that most study centers are tertiary pediatric surgical centers with follow-up care for children with EA and that the parents are used to communicating health topics with their healthcare providers. Yet, healthcare providers should still note that a few individuals may experience the topics as sensitive. The most sensitive items were rated so by only four parents and regarded reactions from other people on their child's condition. Experiences of stigma is reported in children with chronic conditions (64) and adults with EA (65, 66).

Given that the EA-QOL questionnaire only includes 17 items, the results for item comprehensiveness were satisfactory; only a few individuals provided additional suggestions. Although the study protocol instructed researchers to ask for item comprehensiveness during cognitive debriefing, we cannot exclude these results are due to variation of the interviewer performance. Two important notes should be taken. The EA-QOL questionnaire does not measure the perceived impact of associated anomalies, which may be present in 55% of children with EA. Furthermore, items in the domain eating may not suit children with full enteral feeding, a challenge also seen in another eating instrument for children (67). Items of associated anomalies and gastrostomy were sorted out during the item selection process due to poor item performance (35, 36, 51). Methods such as interviews and computer adaptive testing may help to better address these aspects in children with EA (46).

There were no difficulties in translating the response scale of the EA-QOL questionnaire, but due to heterogeneity in the clinical presentation of EA, the use of a “not applicable” response option was discussed. The clinical heterogeneity could be noted, as, for example, a few items in the eating domain received affirmative comments from some parents, while other parents explained that their child did not experience the situation (e.g., vomiting problems). The EA-QOL questionnaire development followed international recommendations for PROMs where the use of “not applicable” response options was described to create problems and possible bias in scoring (30). The use of such a response option can raise challenges (68) as it could offer an easy way for the respondent if they are not entirely sure of the question, want to avoid committing themselves to answer, or when the survey exceeds their motivation/ability to reply (46, 69–71). Given these multiple reasons, “not applicable” may be challenging to interpret (68). If it is treated as “no problem” in score calculations, results could be biased towards “not affected” and lessen the instrument's ability to capture treatment response (30). However, scores will also be biased if the same respondents decide to skip items because an item is not feasible. There are different schools in the psychometric field (46). While the EA-QOL questionnaires have been evaluated using classical test theory (36, 41–43), a newly developed QOL questionnaire for adults with EA in the Netherlands was evaluated using item response theory and enhanced item feasibility by using the “non-applicable” response option (72). The EA-QOL questionnaire was adapted for the group of children with EA, describing in its questionnaire instructions that respondents may skip an item if it is not applicable for them, but ≥70% item responses for scale score calculations are required to ensure trustworthiness of data (36). In comparison, a well-established generic instrument like PedsQL (73) and a condition-specific instrument like CLEFT-Q (74) require ≥50% of completed items.

In our study, the extent of interview data varied between the countries; items commonly received comments from participants from Croatia, France, Hungary, Norway, Spain, South Africa, and especially China and the UK. This may reflect the quality of the translations and the interview (32, 33, 50). The interviewers had varying professional backgrounds and mostly a care and treatment relationship with the child. On the one hand, this may reflect a trustful setting where parents are comfortable discussing their child's HRQOL. On the other hand, parents could feel dependent on their healthcare provider, so they do their best to provide the information they believe this researcher is requiring (75). Parents did provide suggestions for improvement of the translations, which could indicate their degree of openness. Furthermore, as recommended, the cognitive debriefing enabled parents to rate and comment on the EA-QOL questionnaire, providing two sources of information to increase the soundness of the data (33).

### 4.3. Harmonization

We paid great attention to harmonizing the translations of the EA-QOL questionnaire, which is a key objective to ensure intertranslation validity (39, 45). For the UK-US English version as well as the European Spanish-Mexican Spanish version, different translations were first developed for each country (country-specific approach). For the UK and South African English, the same language adaptation approach was used, meaning that a language version of an instrument existed for one country (the UK) and then was adapted for use in a new country (South Africa) (45). For languages employed in different countries, we performed the harmonization after the cognitive debriefing. Interestingly, a large extent of modifications of the translated items in these countries were due to harmonization, but it was balanced against the need for cultural adaptation of the items. Therefore, the US-UK English versions of the EA-QOL questionnaire were equivalent, picking up, e.g., the need to convert the items to statements and using the gender-neutral expression “they” in the items. In contrast, the South African English version kept an interview-based approach with questions using he/she.

Moreover, both Spanish versions of the EA-QOL questionnaire could be made precisely linguistically equivalent. Compared to a previous study of a condition-specific PROM for children and adults with cleft lift and/or palate (76), 40% of the items differed across the three Spanish varieties, Columbian, Chilean, and Spanish (Spain). In the DISABKIDS project for children with chronic conditions (64), a simultaneous approach was uniquely used for developing condition-specific instruments for seven childhood conditions across seven countries (77–79). In this view, our study reflects twelve new linguistic versions of the EA-QOL questionnaire being established after the initial Swedish-German questionnaire. Nevertheless, to the authors’ knowledge, there is not yet another report reflecting the coordination of 14 translations for a child with a rare pediatric surgical malformation.

### 4.4. Study strengths and limitations

The study is strengthened by incorporating perspectives from parents of children with EA from 14 countries on different continents, instrument developers, native experts in the field of EA, and patient stakeholders to establish linguistic versions of a HRQOL instrument for young children with EA. Although a standardized study protocol worked as a basis for our study, flexibility to each study center/country prerequisites of resources, competence, eligible sample sizes, and ethical regulations was required to enhance the study's feasibility. Therefore, the study is weakened by the variation in the time point of the study, translational procedures, recruitment sources, interviewer experience/skills, and location of data collection. Furthermore, there may be differences in socio-economic standards and language use in different geographical areas in one country, a topic, which goes beyond the scope of our study. There are different views on the quantification of qualitative data like parents’ comments on the EA-QOL questionnaire, proposing either that it could enrich the understanding of complex data or that it should be avoided (80). In qualitative terms, a comment from an informant may be of similar importance to comments made by several informants.

Additionally, although we generally complied with the numbers outlined by ISPOR (39), the study samples in cognitive debriefings in individual countries were small. Due to ethical and feasibility reasons, we presented only the study participants’ anatomical subtypes of EA. In comparison, the rates of Gross type B and C are line with other reports (3), but the prevalence of Gross type A, usually a more severe form, was slightly higher (12.4% vs. 7%–8%). This study used convenient and purposive sampling methods stratifying for severity of EA. Additionally, our samples reflect that most study centers are expert centers for caring for children with EA in their respective countries.

Lastly, if and how the cross-cultural equivalence of an HRQOL instrument can be reached has been intensively debated (37, 56), with assumptions that either connotation of diseases is culture-bound or that HRQOL interpreted within a given culture has universal components. The EA-QOL questionnaire has not been evaluated regarding measurement equivalence (38) to understand if it measures the same latent construct in all country-cultural groups of investigation. A cross-cultural field test evaluating validity and reliability of the EA-QOL questionnaire in a larger sample size of children with EA, reaching statistical power for advanced psychometric testing, is needed. Furthermore, this study is limited to present findings regarding the EA-QOL questionnaire for children aged 2–7 (parent-report). The evaluation of the EA-QOL questionnaire for children aged 8–18 is equally important and will therefore be reported separately.

## 5. Conclusions

Cross-culturally, parents of children with EA aged 2–7 from 14 countries, understand the items of the EA-QOL questionnaire easily and do generally not perceive them as sensitive to answer. When poor translations in individual countries/languages were identified, these were improved for clarity and as far as possible the translations were harmonized with each other. One cross-cultural modification to increase this questionnaire's applicability across 14 countries was judged needed, that was to higher the child age of responding to the Social isolation & Stress domain from age 2 to 4 (parent-report). Hence, unique collaborative efforts in the field of EA has helped establish a semantically and conceptually equivalent HRQOL questionnaire for young children with EA, which their parents and clinical stakeholders understand, for use in research and clinical practice across 14 countries. In our experience, the key components to achieving this work were the joint consideration of perspectives given by parents of children with EA, native experts within the specific field of EA, patient stakeholders, and instrument developers. These efforts have set the conditions for a cross-cultural field test of the EA-QOL questionnaire and will open the doors for a new chapter in outcome research, registries, and clinical practice concerning children with EA. In the long-term, this will help increase knowledge of the disease's burden, promote patient-centeredness, exchange of information between nations, and strengthen evidence-based treatments for children born with EA.

## Data Availability

The original contributions presented in the study are included in the article/**Supplementary Material**, further inquiries can be directed to the corresponding author.

## Group members of The international EA-QOL group

Michaela Dellenmark Blom^1,2,3^*, Stefanie Witt^4^, Benjamin Zendejas^5^, Ivana Sabolić^6^, Juan Domingo Porras-Hernandez^7^, Natalie Durkin^8^, Simon Eaton^8^, Kjersti Birketvedt^9^, Alba Sánchez Galán^10^, Katalin Eszter Müller^11, 12, 13^, Anna Rozensztrauch^14^, Tutku Soyer^15^, Siqi Li^16^, Anastasia Fourtaka^17^, Corne de Vos^18^, Graham Slater^19^, Ana Špoljarić^20^, John Bennett^5^, Ragnhild Emblem^21^, Zita Andrásdi^11,^ Robert Smigiel^14^, Dariusz Patkowski^22^, Çiğdem Ulukaya Durakbaşa^23^, Marina Stilinović^6^, Frederic Gottrand^17^, Dora Škrljak Šoša^6^, Tomislav Luetić^6^, Sylwester Gerus^22^, Shen Yang^16^, Yong Zhao^16^, Yichao Gu^16^, Shuangshuang Li^16^ Diego Rodriguez-Alvirde^7^, Orsolya Kadenczki^24^, Miram Pasini^6^, Vuokko Wallace^25, 26^, Anke Widenmann^27^, Feliciana Milagres Sikwete^18^, Jinshi Huang^16, 28^, Leopoldo Martínez Martínez^10^, Kate Abrahamsson^1^, Shawn Izadi^5^, Benno M Ure^29^, Daniel Sidler^18^, Julia H Quitmann^4, 30^†, Jens Dingemann^29^†

†These authors share last authorship

Affiliations

^1^Department of Pediatrics, Institute of Clinical Sciences, University of Gothenburg, Sweden

^2^Department of Pediatric Surgery, Queen Silvia Children’s Hospital, Gothenburg, Sweden

^3^Department of Women's and Children's Health, Karolinska Institutet, Stockholm, Sweden

^4^Department of Medical Psychology, University Medical Center Hamburg-Eppendorf, Hamburg, Germany

^5^Esophageal and Airway Treatment Center, Department of Pediatric General Surgery, Boston Children's Hospital, Harvard Medical School, Boston, MA, United States

^6^Division of Pediatric Surgery Department of Surgery University Hospital Centre Zagreb Zagreb, Croatia

^7^Surgical Division, Hospital para El Niño Poblano, Puebla, México

^8^UCL Great Ormond Street Institute of Child Health, 30 Guilford Street, London WC1N1EH, United Kingdom

^9^Centre of Rare Disorders, Division of Pediatric and Adolescent Medicine, Oslo University Hospital, Oslo, Norway

^10^University Hospital La Paz, Madrid, Spain

^11^Heim Pál National Pediatric Institute, Budapest, Hungary

^12^Institute for Translational Medicine, Faculty of Medicine, University of Pécs, Hungary

^13^Doctoral School of Clinical Medicine, University of Debrecen, Debrecen, Hungary

^14^Department of Nursing and Obstetrics, Division of Family and Pediatric Nursing, Wroclaw Medical University, 50-367 Wroclaw, Poland

^15^Department of Pediatric Surgery, Faculty of Medicine, Hacettepe University, Ankara, Turkey

^16^Department of Neonatal Surgery, Beijing Children’s Hospital, Capital Medical University, National Center for Children’s Health, Beijing 100045, China

^17^Centre Hospitalier Universitaire Hôpital Jeanne de Flandre Pôle Enfant 2 avenue Oscar Lambret - 59037 Lille Cedex

^18^Tygerberg Hospital, Stellenbosch University, South Africa

^19^EAT (Esophageal Atresia Global Support Groups), Sommerrainstr. 61, 70374 Stuttgart, Germany; and TOFS, Nottingham, United Kingdom and TOFS St George’s Centre, Netherfield, Nottingham NG4 2NN, UK

^20^Division of Pediatric Surgery Department of Surgery University Hospital Centre Zagreb Zagreb, Croatia

^21^Department of Paediatric Surgery, Oslo University Hospital, Oslo, Norway

^22^Department of Paediatric Surgery and Urology, Wrocław Medical University, Borowska 213, 50-556 Wrocław

^23^Department of Pediatric Surgery, Faculty of Medicine, Istanbul Medeniyet University, Istanbul, Turkey

^24^Department of Pediatrics, Faculty of Medicine, University of Debrecen, 4032 Debrecen, Hungary

^25^University of Bath, Department of Psychology, Building 10 West, Bath BA2 7AY, UK

^26^EAT (Esophageal Atresia Global Support Groups), Sommerrainstr. 61, 70374 Stuttgart, Germany

^27^EAT (Esophageal Atresia Global Support Groups), Sommerrainstr. 61, 70374 Stuttgart, Germany; and KEKS national support group in Germany, Sommerrainstr. 61, 70374 Stuttgart, Germany

^28^Department of Neonatal Surgery, The Affiliated Children’s Hospital of Nanchang University, Nanchang 330006, China

^29^Centre of Pediatric Surgery, Hannover Medical School, Hannover

^30^Hochschule für Angewandte Wissenschaften Hamburg (HAW Hamburg), Germany
