# Supplementary material for: Establishment of a condition-specific quality-of-life questionnaire for children born with esophageal atresia aged 2–7 across 14 countries
Source: Front Pediatr. 2023 Oct 23;11:1253892. doi: 10.3389/fped.2023.1253892 (PMC10626467; doi:10.3389/fped.2023.1253892)
Supplement: Supplementary file 1 [file Datasheet1.pdf]

Supplementary Material 1

Establishment of a condition-specific quality-of-life questionnaire for children born with esophageal atresia aged 2-7 across 14 countries

The International EA-QOL group

***Correspondence:**
Michaela Dellenmark-Blom, E-mail: michaela.m.blom@vgregion.se

The target languages, year of establishment, key in-country consultants, professionals involved in the forward-backward translation, and result of the back-translation review

The UK and US English translation were completed already in 2015 and in conjunction with the establishment of the study protocol, it was again professionally reviewed by a native English speaker for US and UK English in 2017. The nine additional translations were conducted between 2018-2022. In each country, a key in-country consultant managed the process and was the primary contact person with the instrument developer (MDB). To achieve the forward-backward translation, the research team in four countries used translators in a healthcare profession; in three countries, the team recruited translators from a professional agency, whereas in four countries both options were used. Following two independent forward-translations, reconciliation and a back-translation, the semantical /dis/agreements between the back-translation and source version of the EA-QOL questionnaire were recorded by the instrument developer (MDB) and the key in-country consultant using a standardized excel-file for documentation. In three countries (China, Hungary, Spain), the research team was permitted to use the English version of the EA-QOL questionnaire for translation into the new language, as already described in detail for the Chinese Mandarin language [1]. If the English version was treated as source version, the back translation review was additionally conducted by a native English speaker fluent in Swedish who was recruited for this purpose only. If the back-translation review indicated a need to revise the new translation, it was discussed between the native experts and instrument developer (MDB) until a consensus on a good translation was reached. Throughout the process, the study protocol describing the aim of the items in the EA-QOL questionnaire, item key words and quotes from children and parents from the Swedish focus groups were used to promote conceptual equivalence across all translations.

Supplemental material 1. The target languages, year of establishment, key in-country consultants, actors involved in the forward-backward translation, and result of the back-translation review

**Continent**		**Africa**	**Asia**	**Europe**								**Central-America**	**North-America**
**New languages**	**Target language**	South-African English	Mandarin Chinese	Croatian	French	Hungarian	Norwegian	Polish	Spanish	Turkish	UK English	Mexico	USA
**Source language**	Language used for translation to the target language	Swedish	English	Swedish	Swedish	English	Swedish	Swedish	English	Swedish	Swedish	Swedish	Swedish
Key-in country consultant	Main contact person managing the process in the target country, native in the target language, fluent in the source language	Recruited translator with health care profession who was not part of the research team. From 2019, Corné de Vos.	Siqi Li	Ivana Sabolic	Anastasia Fourtaka	Katalin Eszter Müller	Kjersti Birketvedt	Anna Rozensztrauch	Alba Sánchez Galán	Tutku Soyer	Recruited translator with health care profession who was not part of the research team. From 2019, Natalie Durkin	Juan Domingo Porras-Hernandez	Recruited translator with health care profession who was not part of the research team. From 2018, Benjamin Zendejas Mummert
**Forward-backward translation**	Year of translation	2016	2020	2020	2021	2019	2021	2020	2019	2018	2016	2022	2015-2017
	Translator from professional agency	Yes	No	Yes	Yes	No	No	Yes	Yes	No	Yes	Yes	Yes
	Translator health care professional[a]	Yes	Yes	No	No	Yes	Yes	Yes	No	Yes	Yes	Yes	Yes
**Back-translation review**	Number of items reviewed and solved to establish semantical equivalence with the source language	8	7	3	3	6	3	2	4	1	8	7	8
