# Supplementary material for: Establishment of a condition-specific quality-of-life questionnaire for children born with esophageal atresia aged 2–7 across 14 countries
Source: Front Pediatr. 2023 Oct 23;11:1253892. doi: 10.3389/fped.2023.1253892 (PMC10626467; doi:10.3389/fped.2023.1253892)
Supplement: Supplementary file 2 [file Datasheet2.pdf]

Supplementary Material 2

Establishment of a condition-specific quality-of-life questionnaire for children born with esophageal atresia aged 2-7 across 14 countries

The International EA-QOL group

***Correspondence:**
Michaela Dellenmark-Blom, E-mail: michaela.m.blom@vgregion.se

Description of the data collection method used in the cognitive debriefing interviews of the EA-QOL questionnaire for children aged 2-7 listed in order of the earliest study year and country

Country	Study year	Recruitment source	Number of interviewers	Profession of interviewer	Care and treatment relationship	Location of the interviews
**Africa**						
South Africa	2021	Clinical center	2	Registrar in paediatric surgery and rotating Intern	No	Clinical center/Hospital
**Asia**						
China	2021	Clinical center	1	Master degree candidate in medicine	No	Online/Virtual interviews
**Europe**						
Croatia	2020	Clinical center	3	Medical Doctor<br>Professor of pedagogy<br>Pediatric surgeon	No	Clinical center/Hospital
France	2021-2022	Clinical center	1	Psychologist	Yes	Clinical center/Hospital
Hungary	2020	Clinical center, Patient support group	2	Pediatric gastroenterologist PhD student, a clinical laboratory scientist	No	University
Norway	2021-2022	Clinical center, Patient support group	3	Medical doctors, Reg. Dietician	Yes[1]/No	Clinical center/Hospital, Telephone (two interviews)
Poland	2021	Clinical center	4	Pediatric Nurse, Geneticist, Pediatric Surgeons	Yes	Clinical center/Hospital
Sweden-Germany	2016	Clinical center	3	Pediatric Nurse, PhD student, Psychologist, PhD student	No	Clinical center/Hospital
Spain	2019	Clinical center	1	Pediatric Surgeon	No	Clinical center/Hospital
Turkey	2019	Clinical center	2	Pediatric Surgeons	Yes	Clinical center/Hospital University
United Kingdom	2020	Patient support group, TOFS	1	Pediatric Surgeon	No	Online/Virtual interviews
**Central-America**						
Mexico	2022	Clinical center	2	Pediatric Surgeons	Yes	Clinical center/Hospital
**North-America**						
USA	2020	Clinical center	1	Quality improvement consultant	No	Clinical center/Hospital

[1]The researcher with the main responsibility of the cognitive debriefing interviews and conducted most of them did not have a care and treatment relationship with the children
