# Supplementary material for: Establishment of a condition-specific quality-of-life questionnaire for children born with esophageal atresia aged 2–7 across 14 countries
Source: Front Pediatr. 2023 Oct 23;11:1253892. doi: 10.3389/fped.2023.1253892 (PMC10626467; doi:10.3389/fped.2023.1253892)
Supplement: Supplementary file 3 [file Datasheet3.pdf]

Supplementary Material 3

Establishment of a condition-specific quality-of-life questionnaire for children born with esophageal atresia aged 2-7 across 14 countries

The International EA-QOL group

***Correspondence:**
Michaela Dellenmark-Blom, E-mail: michaela.m.blom@vgregion.se

Supplemental material 3.

Description of item performance from cognitive debriefing of the EA-QOL questionnaire for children with esophageal atresia aged 2-7, conducted with parent-proxies from Europe (Sweden-Germany[1], Croatia, France, Hungary, Norway, Poland, Spain, Turkey, United Kingdom-UK), Africa (South Africa), Asia (China[2]), Central America (Mexico) and North-America (USA)

- Supplemental material 3a. Number and percentage of parents of children with esophageal atresia aged 2-7 who rated the item of the EA-QOL questionnaires as easy to understand (item clarity) in individual countries and cross-culturally

- Supplemental material 3b. Number and percentage of parents of children with esophageal atresia aged 2-7 who rated the item of the EA-QOL questionnaire as sensitive to answer (interferes with personal integrity/privacy)

- Supplemental material 3c. Missing item responses, n % in the EA-QOL questionnaire for children aged 2-7 in individual countries and cross-culturally

Supplemental material 3a. Number and percentage of parents of children with esophageal atresia aged 2-7 who rated the item of the EA-QOL questionnaires as easy to understand (item clarity)

			EUROPE									AFRICA	ASIA	CENTRAL-AMERICA	NORTH-AMERICA	CROSS-CULTURAL
**Domains**		**Items**	**Sweden-Germany (n=16)**	**Croatia (n=14)**	**France (n=6)**	**Hungary (n=9)**	**Norway (n=10)**	**Poland (n=9)**	**Spain (n=8)**	**United Kingdom (n=11)**	**Turkey (n=8)**	**South Africa (n=4)**	**China (n=8)**	**Mexico (n=13)**	**USA(n=13)**	**Cross-cultural (n=113)[f]**
**Eating**	1.	It is difficult for my child to eat age-appropriate food because food sticks in their throat	15(100)[a]	14(100)	6(100)	9(100)	10(100)	9(100)	8(100)	8(72.7)	8(100)	4(100)	8(100)	13(100)	13(100)	110(97.3)
	2	It is difficult for my child to eat a full meal	14(100)[b]	14(100)	6(100)	9(100)	6(60)	9(100)	8(100)	10(90.9)	8(100)	4(100)	8(100)	12(92.3)	11(84.6)	105(92.9)
	3	Eating stresses my child	14(93.3)	14(100)	6(100)	9(100)	8(80)	9(100)	8(100)	10(100)[a]	8(100)	4(100)	8(100)	12(92.3)	13(100)	109(97.3)[a]
	4.	My child can eat at the pace they want	13(92.9) [b]	13(92.9)	6(100)	9(100)	8(80)	9(100)	7(87.5)	9(81.8)	8(100)	4(100)	8(100)	12(92.3)	13(100)	106(93.8)
	5	My child is worried when they choke on food	15(100)[a]	14(100)	6(100)	9(100)	7(70)	9(100)	7(87.5)	9(81.8)	8(100)	4(100)	8(100)	13(100)	10(76.9)	104(92.0)
	6.	It bothers my child when they vomit	16(100)	14(100)	6(100)	9(100)	8(80)	9(100)	6(75.0)	8(72.7)	8(100)	4(100)	8(100)	12(92.3)	12(92.3)	104(92.0)
	7	My child requires certain adaptations so they can eat food at a party or when out with friends	16(100)	14(100)	6(100)	9(100)	8(80)	9(100)	8(100)	9(81.8)	8(100)	4(100)	8(100)	13(100)	12(92.3)	108(95.6)
**Physical health & treatment**	8.	My child gets tired easily when they play games or sports	16(100)	14(100)	6(100)	9(100)	10(100)	9(100)	8(100)	11(100)	8(100)	4(100)	8(100)	13(100)	13(100)	113(100)
	9.	My child has less strength than other children during physically demanding activities	16(100)	14(100)	6(100)	9(100)	9(90)	9(100)	8(100)	11(100)	8(100)	4(100)	8(100)	12(92.3)	11(84.6)	109(96.5)

	10.	My child is bothered by respiratory problems (e.g. coughing, phlegm, or difficulty breathing)	15(100)[a]	14(100)	6(100)	9(100)	8(80)	9(100)	6(75.0)	6(54.5)	8(100)	4(100)	8(100)	13(100)	13(100)	104(92.0)
	11.	It is a problem for my child that my child gets respiratory infections easily	16(100)	14(100)	2(33)	9(100)	8(80)	9(100)	7(87.5)	3(27.3)	8(100)	4(100)	8(100)	12(92.3)	10(76.9)	93(83.0)
	12.	My child hates taking medicine	14(93.3)[a]	14(100)	1(16.7)	9(100)	6(60)	9(100)	8(100)	11(100)	8(100)	4(100)	8(100)	13(100)	13(100)	104(92.0)
	13.	My child's health condition makes it difficult for them to fall asleep or stay asleep at night (e.g. reflux, coughing, anxiety)	16(100)	14(100)	6(100)	9(100)	10(100)	9(100)	8(100)	8(72.7)	8(100)	4(100)	8(100)	13(100)	13(100)	110(97.3)
Social isolation & stress	14	Preschool/school absence due to my child's health condition impacts my child's life negatively	15(100)[a]	12(100)[b]	6(100)	7(100)[b]	5(50)	9(100)	7(87.5)	4(36.4)	8(100)	2(100)[b]	8(100)	13(100)	13(100)	94(87.9)[d]
	15.	It is hard for my child to explain to others what they can and cannot do	13(100)[c]	14(100)	6(100)	6(85.7)[b]	3(30)	9(100)	8(100)	10(100)[a]	8(100)	3(100)[a]	8(100)	13(100)	13(100)	101(92.7)[e]
	16.	It bothers my child that people make comments about them (e.g. coughing, scars, choking)	15(100)[a]	14(100)	6(100)	8(100)[a]	4(40)	9(100)	8(100)	9(90)[a]	8(100)	3(100)[a]	8(100)	13(100)	13(100)	103(93.6)[c]
	17	It bothers my child that people react negatively when they make a noise (e.g. breathing, clearing his/her throat, coughing,, wheezing)	16(100)	14(100)	6(100)	7(100)[b]	4(40)	9(100)	8(100)	11(100)	8(100)	4(100)	8(100)	13(100)	13(100)	105(94.6)[b]
	[a] 1 missing, [b] 2 missing, [c] 3 missing, [d] 6 missing, [e] 4 missing, [f] excluding data from Sweden and Germany															

Supplemental material 3b. Number and percentage of parents of children with esophageal atresia aged 2-7 who rated the item of the EA-QOL questionnaire as sensitive to answer (interferes with personal integrity/privacy)

			EUROPE									AFRICA	ASIA	CENTRAL-AMERICA	NORTH-AMERICA	CROSS-CULTURAL[f]
**Domains**		**Items**	**Swedish-German (n=16)**	**Croatia (n=14)**	**France (n=6)**	**Hungary (n=9)**	**Norway (n=10)**	**Poland (n=9)**	**Spain (n=8)**	**Turkey (n=8)**	**United Kingdom (n=11)**	**South Africa(n=4)**	**China(n=8)**	**Mexico(n=13)**	**USA(n=13)**	**Cross-cultural (n=113)**
**Eating**	1.	It is difficult for my child to eat age-appropriate food because food sticks in their throat	2(13.3)[a]	0	0	0	0	0	0	0	0	0	0	0	1(7.7)	1(0.9)
	2	It is difficult for my child to eat a full meal	2(14.2)[b]	0	0	0	0	0	0	0	0	0	0	0	2(15.4)	2(1.8)
	3	Eating stresses my child	3(18.8)	0	0	0	1(10)	0	0	0	0	1(25.0)	0	0	1(7.7)	3(2.7)
	4.	My child can eat at the pace they want	2(13.3)[a]	0	0	0	0	0	0	0	0	0	0	0	1(7.7)	1(0.9)
	5	My child is worried when they choke on food	3(20.0)[a]	0	0	0	0	0	0	1(12.5)	0	0	0	0	2(15.4)	3(2.7)
	6.	It bothers my child when they vomit	3(18.8)	0	0	0	0	0	0	0	0	1(25.0)	0	0	2(15.4)	3(2.7)
	7	My child requires certain adaptations so they can eat food at a party or when out with friends	1(6.3)	0	0	0	0[a]	1 (11.1)	0	0	2 (18.2)	1(25.0)	0	0	1(7.7)	5(4.5)[a]

Physical health & treatment	8.	My child gets tired easily when they play games or sports	1(6.3)	0	0	0	0	1 (11.1)	0	1(12.5)	0	0	0	0	1(7.7)	3(2.7)
	9.	My child has less strength than other children during physically demanding activities	2(12.5)	0	0	0	1(10)	1 (11.1)	0	1(12.5)	0	0	0	0	0	3(2.7)
	10.	My child is bothered by respiratory problems (e.g. coughing, phlegm, or difficulty breathing)	1(6.3)[a]	0	0	0	1(10)	1 (11.1)	0	0	0	0	0	0	1(7.7)	3(2.7)
	11.	It is a problem for my child that my child gets respiratory infections easily	1(6.3)	0 [a]	0	0	0	0	0	0	0	0	0	0	1(7.7)	1(0.9)[a]
	12.	My child hates taking medicine	0[a]	0	0	0	0	0	0	1(12.5)	0	0	0	0	1(7.7)	2(1.8)[d]
	13.	My child's health condition makes it difficult for them to to fall asleep or stay asleep at night (e.g. reflux, coughing, anxiety)	0	0	0	0	0	0	0	0	0	0	0	0	1(7.7)	1(0.9)
Social isolation & stress	14	Preschool/school absence due to my child's health condition impacts my child's life negatively	1(6.7)[a]	0[b]	1(16.7)	1(14.3)[b]	0 [a]	0	0	0	1(9.1)	0[c]	0	0	0	3(2.9)[e]

	15.	It is hard for my child to explain to others what they can and cannot do	1(7.1)[b]	0	0	1(14.3)[b]	2(20)	0	0	0	0[a]	0[b]	0	0	0	3(2.8)[f]
	16.	It bothers my child that people make comments about them (e.g. coughing, scars, choking)	0[a]	0	0	0[a]	2(20)	0	0	0	1(10)[a]	0[b]	0	0	1(7.7)	4(3.7)[d]
	17	It bothers my child that people react negatively when they make a noise (e.g. breathing, clearing his/her throat, coughing,, wheezing)	3(18.8)	0	0	0[b]	1(10)	0	0	0	1(9.1)	1(25.0)	0	0	1(7.7)	4(3.6)[b]

[a] 1 missing, [b] 2 missing, [c] 3 missing, [d] 4 missing, [e] 8 missing, [f] 5 missing, [f] excluding data from Sweden and Germany

Supplemental material 3c. Missing item responses, n (% cross-culturally) in the EA-QOL questionnaire for children aged 2-7

			EUROPÉ								AFRICA	CENTRAL-AMERICA	NORTH-AMERICA	CROSS-CULTURAL
**Domains**		**Items**	Sweden-Germany (n=15)	Croatia (n=15)	France (n=6)	Hungary (n=9)	Poland (n=9)	Spain (n=8)	Turkey (n=8)	United Kingdom (n=11)	South Africa(n=4)	Mexico (n=13)	USA (n=13)	**Cross-cultural (n=97)[a]**
**Eating**	1.	It is difficult for my child to eat age-appropriate food because food sticks in their throat	0	0	0	0	0	0	0	0	0	0	0	0
	2	It is difficult for my child to eat a full meal	0	0	0	0	0	0	0	1	0	0	0	1(1.0)
	3	Eating stresses my child	0	0	0	0	0	0	0	0	0	0	0	0
	4.	My child can eat at the pace they want	0	0	0	0	0	0	0	0	0	0	0	0
	5	My child is worried when they choke on food	1	0	0	0	0	0	0	0	0	0	0	0
	6.	It bothers my child when they vomit	0	0	0	0	0	0	0	0	0	0	0	0
	7	My child requires certain adaptations so they can eat food at a party or when out with friends	1	0	0	0	0	0	0	1	0	0	0	1(1.0)
**Physical health & treatm**	8.	My child gets tired easily when they play games or sports	0	0	0	0	0	0	0	0	0	0	0	0

	9.	My child has less strength than other children during physically demanding activities	0	0	0	0	0	0	0	0	0	0	0	0
	10.	My child is bothered by respiratory problems (e.g. coughing, phlegm, or difficulty breathing)	1	0	0	0	0	0	0	1	0	0	0	1(1.0)
	11.	It is a problem for my child that my child gets respiratory infections easily	0	0	4	0	0	0	0	1	0	0	0	5(5.2)
	12.	My child hates taking medicine	0	0	4	0	0	0	0	0	0	0	0	4(4.1)
	13.	My child's health condition makes it difficult for them to fall asleep or stay asleep at night (e.g. reflux, coughing, anxiety)	1	0	0	0	0	0	0	0	0	0	0	0
**Social isolation & stress**	14	Preschool/school absence due to my child's health condition impacts my child's life negatively	2	3	1	3	0	0	0	2	3	0	1	13(13.4)
	15.	It is hard for my child to explain to others what they can and cannot do	3	0	2	3	0	0	0	5	2	0	0	12(12.4)
	16.	It bothers my child that people make comments about them (e.g. coughing, scars, choking)	0	0	1	1	0	0	0	4	2	0	0	8(8.2)
	17	It bothers my child that people react negatively when they make a noise (e.g. breathing, clearing his/her throat, coughing,, wheezing)	1	0	0	2	0	0	0	2	0	0	0	4(4.1)

[a] excluding data from Sweden, Germany, Norway and China

References

[1] Dellenmark-Blom M, Abrahamsson K, Quitmann JH, Sommer R, Witt S, Dingemann J, et al. Development and pilot-testing of a condition-specific instrument to assess the quality-of-life in children and adolescents born with esophageal atresia. Diseases of the Esophagus 2017;30(7):1-9

[2] Li S, Dellenmark-Blom M, Zhao Y, Gu Y, Li S, Yang S, Quitmann JH, Huang J. The Chinese Mandarin Version of the Esophageal-Atresia-Quality-of-Life Questionnaires for Children and Adolescents: Evaluation of Linguistic and Content Validity. Int J Environ Res Public Health. 13;19(22):14923. doi: 10.3390/ijerph192214923.
