# Supplementary material for: Establishment of a condition-specific quality-of-life questionnaire for children born with esophageal atresia aged 2–7 across 14 countries
Source: Front Pediatr. 2023 Oct 23;11:1253892. doi: 10.3389/fped.2023.1253892 (PMC10626467; doi:10.3389/fped.2023.1253892)
Supplement: Supplementary file 4 [file Datasheet4.pdf]

Supplementary Material 4

Establishment of a condition-specific quality-of-life questionnaire for children born with esophageal atresia aged 2-7 across 14 countries

The International EA-QOL group

***Correspondence:**
Michaela Dellenmark-Blom, E-mail: michaela.m.blom@vgregion.se

Supplemental material 4.

Categorization of parents' understanding and perceived difficulties of items in the EA-QOL questionnaire for children with esophageal atresia aged 2-7. The categorization is based on their open comments in cognitive debriefing interviews as documented by the interviewer. The number (n) of parents who described understanding or a type of difficulty is presented. While 116 parents of children with EA aged 2-7 rated the items, between 0-12 parents commented on the items in own words.

Supplemental material 4. Parents' (n_{tot}=113) understanding and perceived difficulties of items in the EA-QOL questionnaire for children with esophageal atresia

			**Understanding**		**Item difficulties**												**Suggested improvements**
**Domains**		**Items**	**Recognition and understanding of the situation in context of EA**		**Unclear wording/Ambiguity**		**Difficult to answer if no experience of symptoms/the situation**		**Difficult to answer due to young child age (2-3 year olds)**		**Bothers parents/impact on family rather than or additional to the child**		**Emotive question/strong expression**				
			**n, total**	**Country, number of participants**	**n, total**	**Country, number of participants**	**n, total**	**Country, number of participants**	**n, total**	**Country, number of participants**	**n, total**	**Country, number of participants**	**n, total**	**Country, number of participants**			
Eating	1.	It is difficult for my child to eat age-appropriate food because food sticks in their throat	10	China (n=6)<br>United Kingdom (n=2)<br>Turkey (n=1)<br>Hungary (n=1)	3	United Kingdom (n=4)<br>Croatia (n=1)	1	Croatia (n=1)	0		2	United Kingdom (n=2)<br>China (n=1)	0				United Kingdom (n=2), incorporated<br>China (n=1), incorporated
	2	It is difficult for my child to eat a full meal	6	China (n=5)<br>South Africa (n=1)	4	Norway (n=3)<br>United Kingdom (n=1)	2	Croatia (n=1)<br>Mexico (n=1)	2	Norway (n=2)	3	China (n=3)	0				
	3	Eating stresses my child	7	China (n=7)	2	Turkey (n=1)<br>Mexico (n=1)	2	Croatia (n=1)<br>China (n=1)	1	Norway (n=1)	1	United Kingdom (n=1)	0				United Kingdom (n=1)<br>Mexico (n=1), incorporated
	4.	My child can eat at the pace they want	12	China (n=8)<br>South Africa (n=2)<br>Hungary (n=1)<br>United Kingdom (n=1)	9	Croatia (n=2)<br>United Kingdom (n=1)<br>Spain (n=1)<br>Norway (n=1)<br>Mexico (n=1)	1	Croatia (n=1)	2	Norway (n=2)	0		0				United Kingdom (n=2)<br>Mexico (n=1), incorporated
	5	My child is worried when they choke on food	3	South Africa (n=2)<br>United Kingdom (n=1)	11	China (=8)<br>United Kingdom (n=1)<br>Norway (n=2)	4	Hungary (n=3)<br>Croatia (n=1)	1	Norway (n=1)	0		0				China (n=8), incorporated<br>United Kingdom (n=1)<br>Spain (n=1)<br>Norway (n=2), incorporated
	6.	It bothers my child when they vomit	12	China (n=8)<br>United Kingdom (n=2)<br>Norway (n=1)<br>South Africa (n=1)	9	United Kingdom (n=5)<br>Hungary (n=1)<br>Croatia (n=1)<br>Norway (n=1)<br>Mexico (n=1)	11	Hungary (n=4)<br>United Kingdom (n=3)<br>Turkey (n=1)<br>Croatia (n=1)<br>Spain (n=2)	0		0		0				
	7	My child requires certain adaptations so they can eat food at a party or when out with friends	11	China (n=8)<br>South Africa (n=2)<br>Poland (n=1)	1	Croatia (n=1)	2	Poland (n=1)<br>Croatia (n=1)	1	Norway (n=1)	1	Spain (n=1)	0				United Kingdom (n=1), incorporated
Physical health & Treatment	8.	My child gets tired easily when they play games or sports	8	China (n=8)	0		2	Poland (n=1)<br>Croatia (n=1)	0		0		0				United Kingdom, n=1
	9.	My child has less strength than other children during physically demanding activities	9	China (n=7)<br>Poland (n=1)<br>South Africa (n=1)	2	China (n=1)<br>Mexico (n=1)	1	Croatia (n=1)	1	United Kingdom (n=1)	0		0				United Kingdom, n=1<br>Mexico, n=1, incorporated
	10.	My child is bothered by respiratory problems (e.g. coughing, phlegm, or difficulty breathing)	12	China (n=8)<br>Poland (n=1)<br>Hungary (n=1)<br>South Africa (n=1)<br>United Kingdom (n=1)	8	United Kingdom (n=5)<br>Spain (n=2)<br>Croatia (n=1)	2	Croatia (n=1)<br>Hungary (n=1)	0		0		0				
	11.	It is a problem for my child that they get respiratory infections easily	9	China (n=8)<br>Croatia (n=1)	7	United Kingdom (n=4)	2	Croatia (n=1)<br>Hungary (n=1)	0		3	United Kingdom (n=1)	0				United Kingdom (n=1), incorporated

						France (n=3)						South Africa (n=1)<br>France (n=1)			France (n=2), incorporated<br>Spain (n=1)<br>Mexico (n=1)
	12.	My child hates taking medicine	10	China (n=8)<br>Hungary (n=1)<br>United Kingdom (n=1)	9	France (n=3)<br>China (n=2)<br>Norway (n=4)	1	Croatia (n=1)	0		0		0		France (n=3), incorporated
	13.	My child's health condition makes it difficult for them to fall asleep or stay asleep at night (e.g. reflux, coughing, anxiety)	10	China (n=8)<br>Hungary (n=2)<br>United Kingdom (n=1)	1	United Kingdom (n=1)	1	Croatia (n=1)	0		0		0		United Kingdom (n=3), incorporated
Social isolation & Stress	14.	Preschool/school absence due to my child's health condition impacts my child's life negatively	2	China (n=2)	9	United Kingdom (n=6)<br>Croatia (n=1)<br>Spain (n=1)<br>Norway (n=1)	16	China (n=6)<br>Croatia (n=4)<br>Hungary (n=3)<br>South Africa (n=3)	5	United Kingdom (n=3)<br>Norway (n=2)	2	France (n=2)	0		United Kingdom (n=1)<br>Norway (n=2)
	15.	It is hard for my child to explain to others what they can and cannot do	7	South Africa (n=1)<br>China (n=6)	1	China (n=1)	0		13	Hungary (n=3)<br>Norway (n=4)<br>United Kingdom (n=3)<br>South Africa (n=2)<br>Croatia (n=1)	1	China (n=1)	0		
	16.	It bothers my child that people make comments about them (e.g. coughing, scars, choking)	4	China (n=3)<br>South Africa (n=1)	0		5	China (n=5)	13	Hungary (n=3)<br>Norway (n=4)<br>Croatia (n=2)<br>United Kingdom (n=2)<br>Spain (n=1)<br>South Africa (n=1)	2	United Kingdom (n=2)	0		United Kingdom (n=2), incorporated
	17	It bothers my child that people react negatively when they make a noise (e.g. breathing, clearing his/her throat, coughing,, wheezing)	3	Hungary (n=1)<br>China (n=1)<br>South Africa (n=1)	2	Turkey (n=1)<br>United Kingdom (n=1)	1	China (n=1)	6	Hungary (n=3)<br>Norway (n=2)<br>Croatia (n=1)	4	United Kingdom (n=1)<br>China (n=3)	2	United Kingdom (n=2)	United Kingdom (n=2), incorporated<br>Spain (n=1)
