# Supplementary material for: Establishment of a condition-specific quality-of-life questionnaire for children born with esophageal atresia aged 2–7 across 14 countries
Source: Front Pediatr. 2023 Oct 23;11:1253892. doi: 10.3389/fped.2023.1253892 (PMC10626467; doi:10.3389/fped.2023.1253892)
Supplement: Supplementary file 5 [file Datasheet5.pdf]

Supplementary Material 5

Establishment of a condition-specific quality-of-life questionnaire for children born with esophageal atresia aged 2-7 across 14 countries

The International EA-QOL group

***Correspondence:**
Michaela Dellenmark-Blom, E-mail: michaela.m.blom@vgregion.se

Supplemental material 5

Description of implications of cognitive debriefing, review by experts and patient stakeholders and harmonization of the EA-QOL questionnaire in individual countries

As shown in Table 6, in four countries (Turkey, Poland, Hungary, Croatia), no changes in wording of the translated items were judged needed. In the French translation two items (items 11 and 12), in the Norwegian three items (items 2, 5, 6), in the Chinese Mandarin three items (items 1, 5, 7) were corrected for clarity. The most work to improve clarity regarded the English translation of the EA-QOL questionnaire. Following the cognitive debriefing results, discussion between native experts in UK, US and South Africa (ND, SE, BZ, CdV), patient representative native in English (GS) and the instrument developer (MDB), the following was concluded. All questions were converted into statements, and “he/she” was replaced with a gender-neutral term “they” to improve item clarity in the UK and US English translation. Moreover, seven items in the UK and US English translation (items 1, 6, 7, 13, 14, 16 and 17) were improved in wording to increase clarity. In South Africa, all items but one (item 7) were modified in wording in agreement with changes of the UK and US English in order to increase harmonization of the English translation. However, the native experts in South Africa judged it necessary to keep an interview-based approach with questions asked to the parent to suit the general educational and vocabulary level in the South African population. Moreover, the wording using “he/she” was remained in the questions, as “they” was rarely applied in the South African English everyday language. Additionally, clarifications of two items were made by adapting a simpler wording of items 2 and 3 and in four items, and examples (items 4, 9, 10 and 11).

After cognitive debriefing, the three languages who used the English version for translation into their languages compared their version with English version again. This did not result in any need for changes in the Hungarian or the Chinese Mandarin version of the EA-QOL questionnaire. The European Spanish and Mexican Spanish version were compared with each other and the final US English version, with discussions between the native experts (AGS, JDHP), a bilingual Spanish-English speaker (BZ) and the instrument developer (MDB). Given the cognitive debriefing results, expert review and harmonization, most changes in item wording were made because of the harmonization (European Spanish items 1, 3, 4, 5, 6, 9, 11, 15, 6; Mexican Spanish 1, 2, 5, 8, 10, 11, 13, 15, 16), thereby an equivalent European Spanish-Mexican Spanish version was developed.

Cross-culturally, it was ensured that questionnaire instructions in all languages clearly stated that the parent may skip an item e.g., when an item is difficult to understand and/or does not apply to their child. Additionally, the questionnaire instructions in the Norwegian version were shortened in response to parents' preferences. In response to item feasibility of the Social isolation and stress domain, it was decided that parents of children with EA aged 2-3 years old should respond the two domains "Eating" and "Physical Health & Treatment", which together will form the total EA-QOL scores and parents of children with EA aged 4-7 will respond to three domains "Eating", "Physical Health & Treatment" and "Social isolation and stress", which will together form the total EA-QOL scores of this age group. These recommendations will apply for future use in individual countries and in international research of the EA-QOL questionnaire following a cross-cultural field test, to enhance generalizability of the study results.
